# Identification of Sumoylated Proteins in the Silkworm *Bombyx mori*

**DOI:** 10.3390/ijms151222011

**Published:** 2014-12-01

**Authors:** Xudong Tang, Xuliang Fu, Bifang Hao, Feng Zhu, Shengyan Xiao, Li Xu, Zhongyuan Shen

**Affiliations:** 1 School of Biotechnology, Jiangsu University of Science and Technology, Zhenjiang 212018, China; E-Mails: jtv215@126.com (X.F.); bfhao@just.edu.cn (B.H.); zf200537@163.com (F.Z.); shengyanxiao1989@126.com (S.X.); zjcysxl@163.com (L.X.); szysri@163.com (Z.S.); 2 Department of Silkworm Pathology, Sericultural Research Institute, Chinese Academy of Agricultural Sciences, Zhenjiang 212018, China

**Keywords:** SUMO, SUMOylation, *Bombyx mori*, LC–ESI-MS/MS, baculovirus

## Abstract

Small ubiquitin-like modifier (SUMO) modification (SUMOylation) is an important and widely used reversible modification system in eukaryotic cells. It regulates various cell processes, including protein targeting, transcriptional regulation, signal transduction, and cell division. To understand its role in the model lepidoptera insect *Bombyx mori*, a recombinant baculovirus was constructed to express an enhanced green fluorescent protein (eGFP)-SUMO fusion protein along with ubiquitin carrier protein 9 of *Bombyx mori* (BmUBC9). SUMOylation substrates from *Bombyx mori* cells infected with this baculovirus were isolated by immunoprecipitation and identified by LC–ESI-MS/MS. A total of 68 candidate SUMOylated proteins were identified, of which 59 proteins were functionally categorized to gene ontology (GO) terms. Analysis of kyoto encyclopedia of genes and genomes (KEGG) pathways showed that 46 of the identified proteins were involved in 76 pathways that mainly play a role in metabolism, spliceosome and ribosome functions, and in RNA transport. Furthermore, SUMOylation of four candidates (polyubiquitin-*C*-like isoform X1, 3-hydroxyacyl-CoA dehydrogenase, cyclin-related protein FAM58A-like and GTP-binding nuclear protein Ran) were verified by co-immunoprecipitation in *Drosophila* schneide 2 cells. In addition, 74% of the identified proteins were predicted to have at least one SUMOylation site. The data presented here shed light on the crucial process of protein sumoylation in *Bombyx mori*.

## 1. Introduction

Post-translational modifications (PTMs) involve the addition of a chemical group to the protein after it has been generated by the translational machinery. PTMs are essential for a variety of cellular processes and provide an important type of post-translational regulation. A large number of PTMs such as phosphorylation [1], methylation [2], acetylation [3], and glycosylation [4] are known and they regulate various *Biol.* processes such as transcriptional regulation [5] and protein degradation [6]. Small ubiquitin-like modifier (SUMO) modification (SUMOylation), another type of PTM, is structurally related to ubiquitin. The 3-D structure of the human small ubiquitin-like modifier 1 (SUMO-1) protein is very similar to that of ubiquitin and in several instances SUMO competes with ubiquitin for a given lysine acceptor site, thereby preventing its subsequent poly-ubiquitination and degradation by the proteasome [7,8]. SUMO is believed to play roles in various cellular processes, including protein interaction, subcellular localization, and transcriptional regulation [9,10].

The conjugation of SUMO to target proteins is a cascade of enzymatic reactions: first, the SUMO proteins are processed by the SUMO protease that cleaves the *C* terminus of the nascent SUMO to expose the *C*-terminal di-glycine residue [11,12]; second, the exposed *C*-terminal residue forms a thioester bond with the activating enzymes SUMO-1 activating enzyme subunit 1(SAE1) (Aos1 in yeast) and SAE2 (Uba2 in yeast) in an adenosine-triphosphate (ATP)-dependent manner [13]; then the activated SUMO is transferred to the SUMO conjugating enzyme ubiquitin carrier protein 9 (UBC9) [7]; finally, the SUMO protein forms an isopeptide bond with substrate proteins, a reaction catalyzed by SUMO ligases such as protein inhibitor of activated STAT (PIAS), Ran-bingding protein 2 (RanBP2), or human PcG protein Pc2 [14,15]. It is noteworthy that sumoylation is also a dynamic progress. The SUMO protein can be cleaved from the substrates by the same proteases that process the SUMO precursor. Indeed, it has been reported in *Saccharomyces cerevisiae* that the balance between SUMO conjugation and deconjugation is critical for the normal growth of cells [16,17].

SUMO proteins are highly conserved in a large number of species and have been shown to be important in many eukaryotic cell processes. In humans, at least four SUMO proteins have been found, with 44% amino acid identity between SUMO-1 and SUMO-2/3 and 86% amino acid identity between the closely related SUMO-2 and SUMO-3. It has been reported that the Nematode SUMO protein shows greater similarity to the vertebrate SUMO-1 protein [18], while the SUMO protein from *Drosophila* appears to be more similar to the vertebrate SUMO-2/3 proteins [19]. The genome of the silkworm *Bombyx mori* has one copy of the *sumo* gene; the small ubiquitin-like modifier of *Bombyx mori* (BmSUMO) protein shows greater similarity to the vertebrate SUMO-2/3 proteins, sharing 67% identity with human SUMO-2/3 and 61% identity with human SUMO-4, but only 51.6% identity with human sumo-1.

In *B. mori*, it has been reported that BmSUMO participates in the immune response by regulating the expression of v-rel avian reticuloendotheliosis viral oncogene homolog A of *Bombyx mori* (BmRelA) [20]. However, several other functions of BmSUMO and the identity of the other substrate proteins that are targeted by the SUMOylation system of *B. mori* remain largely unknown. Here, a proteomic approach, based on immunoprecipitation, is reported for identification of substrates modified by SUMOylation, which sheds light on the crucial process of protein sumoylation in *B. mori*.

## 2. Results and Discussion

### 2.1. Results

#### 2.1.1. Subcellular Localization of Small Ubiquitin-Like Modifier of *Bombyx mori* (BmSUMO)

**Figure 1:**
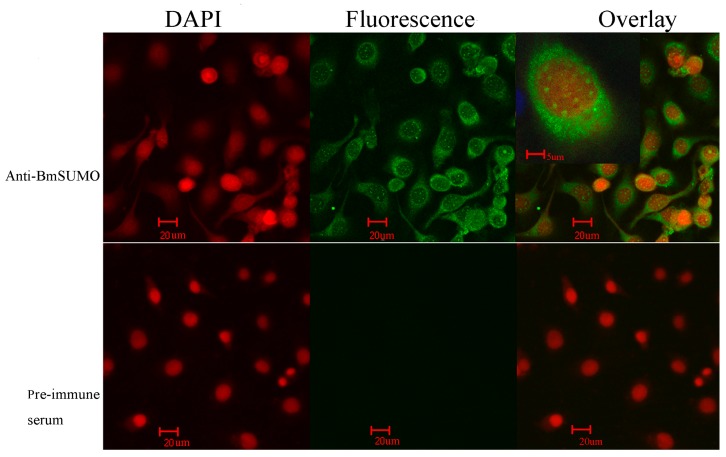
The subcellular localization of small ubiquitin-like modifier of *Bombyx mori* (BmSUMO) in BmN cells. The cells were treated with anti-BmSUMO antibody, and the fluorescent signal was developed by incubating the cells with Protein G fused with enhanced green fluorescent protein. As a control, pre-immune serum was used as the primary antibody. The nuclei were stained with 4',6-diamidino-2-phenylindol (DAPI). The samples were viewed using a confocal laser fluorescence microscope. (Scale bars = 20 and 5 µm respectively).

It has been reported that in the HeLa cell line, SUMO-1, SUMO-2, and SUMO-3 proteins are all localized in the nuclear membrane, in the nuclear bodies, and in the cytoplasm [4,21]. To investigate the subcellular localization of SUMO in BmN cells, immunofluorescence analysis was carried out using a confocal laser-scanning microscope. Although fluorescence was observed across the entire cell, it was mainly observed in the cytoplasm and appeared as aggregated dots in the nucleus (Figure 1). The latter observation was in accordance with that of a previous study that found that many SUMOylated proteins are located in the nucleus [22]. Additionally, SUMO is found to be enriched in the nuclei of cultured *Drosophila* S2 and pole cells, and repression of SUMOylation alters its distribution into the cytoplasm [23,24]. In the control set, no fluorescence was detected (Figure 1).

#### 2.1.2. Isolation of SUMOylated Proteins

**Figure 2:**
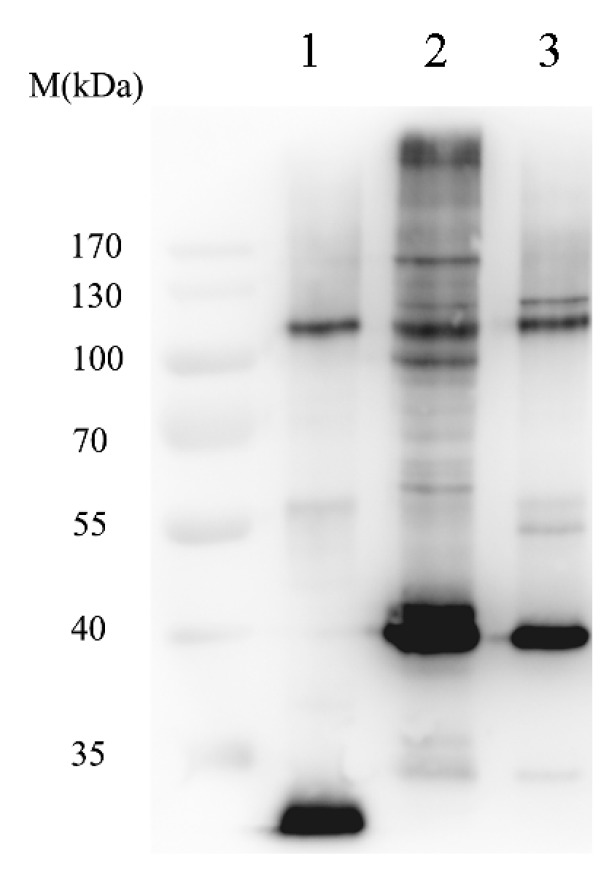
Isolation of SUMO conjugates by immunoprecipitation. Enhanced green fluorescent protein (eGFP), eGFP-SUMO and ubiquitin carrier protein 9 of *Bombyx mori* (BmUBC9), and eGFP-SUMO were expressed in baculovirus vectors and subjected to GFP immunoprecipitation. The immunoprecipitated proteins were then separated by SDS-PAGE and subjected to western blot analysis with an anti-eGFP antibody. Lane **1**, eGFP; Lane **2**, eGFP-SUMO and BmUBC9; Lane **3**, eGFP-SUMO only.

To identify the SUMOylated (modified by SUMO protein) proteins of *B. mori*, a baculovirus vector, which expresses the fusion protein of enhanced green fluorescent protein (eGFP)-sumo and the ubiquitin carrier protein 9 of *Bombyx mori* (BmUBC9), was constructed. BmN cells were infected with the recombinant virus. The SUMOylated proteins were isolated with anti-GFP microbeads. After elution with the supplied elution buffer, the eluate was subjected to SDS-PAGE, followed by western blotting with an anti-GFP antibody. The control was prepared by using the vector expressing eGFP only, and an identical isolation process was performed in parallel. As shown in Figure 2, only the band of eGFP was observed in the control set, while a number of distinct bands, besides that of the 37-kDa eGFP-Bmsumo fusion protein, were observed in the eluate from cells infected with eGFP-SUMO and BmUBC9-expressing recombinant virus. This observation strongly indicated that several SUMOylated candidates had been successfully isolated.

#### 2.1.3. Identification of Sumoylated Proteins by LC–ESI-MS/MS

The isolated SUMOylated proteins from eGFP-SUMO and BmUBC9 preparation were identified by LC–ESI-MS/MS. The eluted proteins were electrophoresed for a short time on SDS-PAGE, following which the bands were cut and processed for LC–ESI-MS/MS analysis. The same protocol was used for the control sample. In total, 68 proteins that were identified by two or more peptides in the eGFP-SUMO and BmUBC9 preparation, and absent from eGFP control preparation (Table 1) were considered candidates for SUMOylation. It should be noted that the control eGFP and eGFP-SUMO preparations have some overlapping proteins, which may have been observed owing to nonspecific interactions. In addition, some proteins were found to be unique to the control sample and may have been purified owing to the complexity of the sample preparation as well as the limitedsequencing time in the mass spectrometer [25]. This possibility was partially confirmed with the MS results of the SUMO proteins present in both control eGFP and eGFP-SUMO preparations; hence, these were excluded from Table 1. Data regarding the proteins identified by a single peptide are presented in Table S1.

**Table 1:** Proteins identified as small ubiquitin-like modifier (SUMO) modification (SUMOylation) substrates.

Protein ID	Description	Number of Peptides
BGIBMGA000115-PA	BUD13 homolog	2
BGIBMGA000511-PA	3-hydroxyacyl-CoA dehydrogenase	5
BGIBMGA000828-PA	low quality protein: DNA replication licensing factor Mcm6-like	2
BGIBMGA001206-PA	chaperonin containing t-complex polypeptide 1 beta subunit	2
BGIBMGA001241-PA	heat shock protein 75 kDa, mitochondrial-like	3
BGIBMGA001549-PA	predicted: polyubiquitin-C-like isoform X1	3
BGIBMGA001627-PA	hypothetical protein KGM_01391	2
BGIBMGA002186-PA	thiol peroxiredoxin	2
BGIBMGA002620-PA	predicted: sequestosome-1-like isoform X2	3
BGIBMGA002755-PA	SUMO-1 activating enzyme	13
BGIBMGA003351-PA	minichromosome maintenance complex component 7	2
BGIBMGA003361-PA	predicted: low quality protein: importin-5-like	2
BGIBMGA003901-PA	predicted: ATP synthase subunit beta, mitochondrial-like	6
BGIBMGA004023-PA	predicted: cyclin-related protein FAM58A-like	12
BGIBMGA004614-PA	predicted: heat shock protein 70 A2-like	6
BGIBMGA004741-PA	predicted: HIV Tat-specific factor 1 homolog	4
BGIBMGA005315-PA	predicted: 26S proteasome non-ATPase regulatory subunit 2-like	2
BGIBMGA005425-PA	ubiquitin-conjugating enzyme E2	23
BGIBMGA005684-PA	60S ribosomal protein L38	4
BGIBMGA005928-PA	ribosomal protein L35	3
BGIBMGA006462-PA	eIF2B-alpha protein	2
BGIBMGA006751-PA	GTP-binding nuclear protein Ran	5
BGIBMGA006980-PA	predicted: nucleolar protein 12-like	3
BGIBMGA007311-PA	40S ribosomal protein SA	3
BGIBMGA007460-PA	predicted: serine/threonine-protein phosphatase 2A catalytic subunit beta isoform-like	3
BGIBMGA007477-PA	receptor for activated protein kinase C RACK 1 isoform 1	4
BGIBMGA007502-PA	predicted: NAD(P) transhydrogenase, mitochondrial-like isoform X1	3
BGIBMGA007720-PA	serine protease inhibitor 2	4
BGIBMGA008295-PA	vacuolar ATP synthase catalytic subunit A	2
BGIBMGA008555-PA	AMP dependent coa ligase	2
BGIBMGA009250-PA	predicted: cAMP-responsive element-binding protein-like 2-like	3
BGIBMGA009816-PA	low quality protein: pre-mRNA-processing-splicing factor 8-like	2
BGIBMGA010005-PA	predicted: transcription initiation factor TFIID subunit 3-like	2
BGIBMGA010361-PA	muscle glycogen phosphorylase	2
BGIBMGA012102-PA	predicted: mediator of RNA polymerase II transcription subunit 10-like isoform X1	5
BGIBMGA012116-PA	predicted: SUMO-activating enzyme subunit 2-like	17
BGIBMGA012126-PA	predicted: eukaryotic translation initiation factor 3 subunit L	2
BGIBMGA012558-PA	predicted: protein timeless homolog	2
BGIBMGA012935-PA	clathrin heavy chain	12
BGIBMGA012976-PA	predicted: serine/threonine-protein kinase TAO3-like	3
BGIBMGA013021-PA	fructose 1,6-bisphosphate aldolase	22
BGIBMGA013063-PA	predicted: malate dehydrogenase, mitochondrial-like	8
BGIBMGA013133-PA	predicted: asparagine—tRNA ligase, cytoplasmic-like	5
BGIBMGA013536-PA	DnaJ-5	3
BGIBMGA013792-PA	40S ribosomal protein S11	5
BGIBMGA014087-PA	peroxiredoxin	3
BGIBMGA014177-PA	predicted: 26S protease regulatory subunit 8-like	3
BGIBMGA014589-PA	phosphate transport protein	2
Bm_nscaf2511_205	predicted: UPF0396 protein CG6066-like	2
Bm_nscaf2681_31	predicted: long-chain-fatty-acid—CoA ligase 4-like isoform X1	3
Bm_nscaf2810_03	hypothetical protein KGM_10301	3
Bm_nscaf2825_24	predicted: la-related protein 7-like	3
Bm_nscaf2829_166	predicted: low quality protein: myosin heavy chain, non-muscle-like	3
Bm_nscaf2839_32	predicted: U4/U6.U5 tri-snRNP-associated protein 1	2
Bm_nscaf2847_240	acinus	2
Bm_nscaf2887_111	predicted: ATP-dependent RNA helicase DDX3X isoform X1	10
Bm_nscaf2888_123	heterogeneous nuclear ribonucleoprotein A1	2
Bm_nscaf2916_07	predicted: putative U5 small nuclear ribonucleoprotein 200 kDa helicase-like	4
Bm_nscaf2930_118	predicted: ADP-ribosylation factor 2-like	2
Bm_nscaf2970_069	mitochondrial matrix protein p32	2
Bm_nscaf2970_070	predicted: zinc finger CCHC domain-containing protein 4-like isoform X1	6
Bm_nscaf2993_193	predicted: striatin-3-like isoform X1	2
Bm_nscaf3015_085	CAAX prenyl protease 1 homolog	3
Bm_nscaf3027_031	asparagine synthetase	2
Bm_nscaf3031_268	DNA topoisomerase 2	6
Bm_nscaf481_60	protein LTV1 homolog	2
BGIBMGA010396-PA	arginine/serine-rich splicing factor 7	2
BGIBMGA010668-PA	predicted: barrier-to-autointegration factor B-like	3

#### 2.1.4. Confirmation of SUMOylated Proteins by Co-Immunoprecipitation (co-IP)

**Figure 3:**
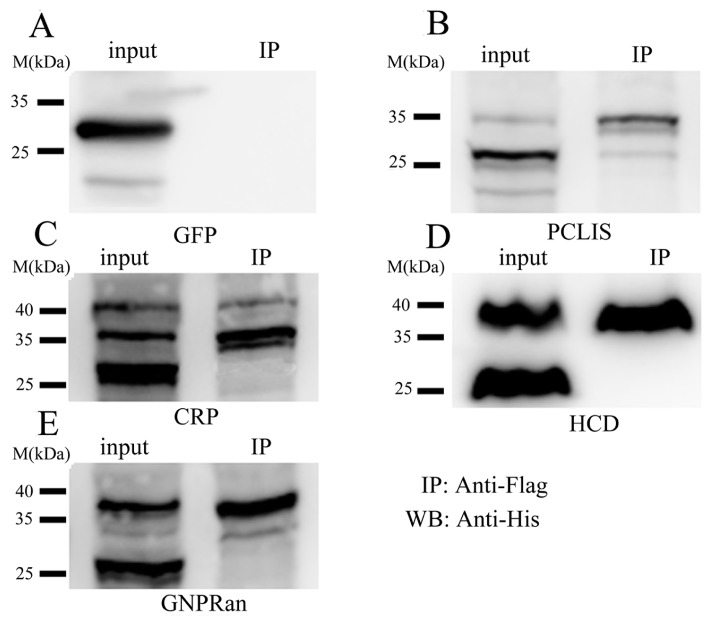
Confirmation of SUMOylated proteins by co-immunoprecipitation (co-IP). Flagged-SUMO and His-tagged polyubiquitin-*C*-like isoform X1 (PCLIS), 3-hydroxyacyl-CoA dehydrogenase (HCD), cyclin-related protein FAM58A-like (CRP) and GTP-binding nuclear protein Ran (GNPRan) were co-expressed in *Drosophila* S2 cells. Cell lysates were immunoprecipitated with anti-Flag magnetic beads and analyzed by western blotting using anti-His antibody. (**A**) GFP; (**B**) PCLIS; (**C**) CRP; (**D**) HDC; (**E**) GNPRan.

To validate our proteomics approach, four candidate proteins such as BGIBMGA001549 (polyubiquitin-*C*-like isoform X1, PCLIS), BGIBMGA000511 (3-hydroxyacyl-CoA dehydrogenase, HCD), BGIBMGA004023 (cyclin-related protein FAM58A-like, CRP) and BGIBMGA006751 (GTP-binding nuclear protein Ran, GNPRan) were selected to verify their modification by SUMO using co-immunoprecipitation (co-IP). Flag-tagged SUMO and His-tagged candidates were co-expressed in *Drosophila* S2 cells. Two days after inducing expression by CuSO_4_. Cells were processed for immunoprecipitation with anti-Flag magnetic beads and analyzed by western blotting using anti-His antibody. As shown in Figure 3, in all four of these cases examined, His-tagged candidate proteins could be immunoprecipitated from candidate proteins + SUMO transfected S2 cells; the apparent molecular weights are compatible with attachment of one (Figure 3B,D,E) or two SUMO proteins (Figure 3C). No proteins were observed in the control set of GFP + SUMO transfected S2 cells.

#### 2.1.5. Functional Annotation of sumoylated Proteins

**Figure 4:**
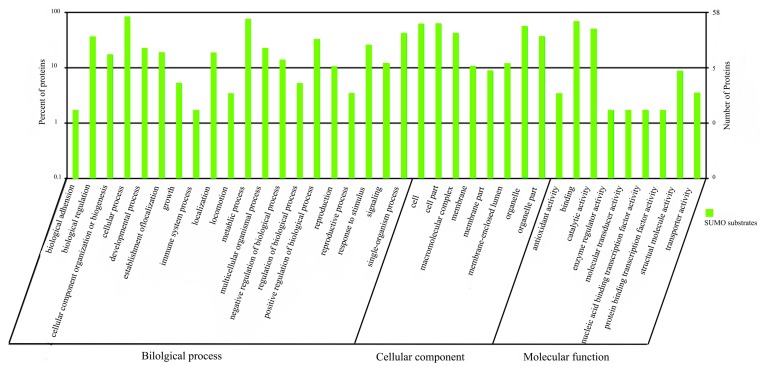
Gene ontology (GO) categories of SUMO-conjugated proteins. Proteins were classified into *Biol.* process, cellular component, and molecular function categories by Web Gene Ontology Annotation Plot (WEGO), according to the GO hierarchy. The number of proteins mapped to the GO terms is shown on the right axis. The left axis shows the proportion of total proteins mapped to the GO terms.

To understand the functions of the identified proteins, the protein sequences were queried against the InterPro databases. The resultant proteins were functionally categorized based on universal gene ontology (GO) annotation terms by using the online GO tool WEGO (Web Gene Ontology Annotation Plot) and were classified into cellular component, molecular function, and biological process categories, according to the GO hierarchy by using WEGO. The majority of the proteins (59 of 68) were functionally categorized to GO terms (Figure 4). Under the “biological process” category, the proteins were involved in “cellular process” (19.39%), “metabolic process” (16.48%), “single organism process” (9.2%), and “biological regulation” (8.05%). Under the “cellular component” category, the proteins were classified as “cell” (21.56%), “cell part” (21.56%), “organelle” (16.5%) and “macromolecular complex” (14.37%). Under the “molecular function” category, the identified proteins were mainly involved in “binding” (48.15%) and “catalytic activity” (35.8%) (Figure 4).

Analysis by the kyoto encyclopedia of genes and genomes (KEGG) tool showed that 46 of the identified proteins were involved in 76 pathways, which were largely classified into metabolism, genetic information processing, environmental information processing, cellular processes, organismal systems, and human disease. Pathways that play a role in metabolism, spliceosome and ribosome functions, and in RNA transport showed the highest number of matches.

### 2.2. Discussion

Being an important PTM, sumoylation regulates many cellular pathways. However, identifying new SUMO targets and understanding the functions of protein SUMOylation has been largely limited by the low level of SUMOylation. It has been reported that the SUMO-Ubc9 fusion protein has a higher conjugating activity for SIM-containing targets such as Sp100 and human cytomegalovirus immediate-early 2 (IE2) protein [26]. Hence, we used a special baculovirus vector, which can express foreign proteins under different promoters, to express eGFP-sumo and BmUbc9. The control construct that could express eGFP-SUMO, but lacked BmUbc9, was also used for immunoprecipition (Figure 2, lane 3). The data presented here show that compared to eGFP-sumo, eGFP-sumo and BmUbc9 bound to a much larger number of diverse cellular proteins and interacted with some SIM-containing proteins with higher affinities. Thus, the results confirmed that the SUMO-Ubc9 fusion construct could be useful for identifying or purifying SUMO-modified proteins and for modulating the cellular SUMO pathway. However, it should be noted that over-expression of both SUMO and Ubc9 may lead to the pull-down of contaminants, non-relevant SUMO targets or proteins interacting non-covalently with SUMO (but not SUMOylated themselves). Of the 68 candidate SUMOylated proteins, some may not be truly modified by SUMOylation *in vivo*. Additional experiments such as immunoblotting and/or SUMOylation site identifications should be performed to confirm the status and dynamics of SUMOylation of each individual target.

A number of proteomic studies have been performed to identify the substrates of SUMOylation in both yeast and higher eukaryotes [27,28,29,30]. On comparing the potential substrates of silkworm with the yeast data [25], some homologs such as budding proteins (Buds), transcriptional activator Gcns Uba2, and transcript elongation factors (Spts) were identified. In this study, all the components of the multiprotein complex involved in sumoylation, including SAE1, SAE2, and PIAS, were found to coimmunoprecipitate in the presence of eGFP-Bmsumo and Bmubc9. Furthermore, BmUBC9 and BmSUMO were identified in both eGFP and eGFP-sumo plus BmUbc9 preparations (data not shown). Some of the homologous proteins identified in this study have been previously reported to be SUMOylated. For example, the endoplasmic reticulum-resident chaperone calreticulin is known to be sumoylated, and abolishing sumoylation enhanced calreticulin expression in an X Box binding protein 1 (XBP-1) dependent manner, resulting in increased calreticulin-counteracted endoplasmic reticulum stress [31]. The human transcription factor II D (TFIID) subunits TBP-associated factors 5 (TAF5) and TAF12 are modified by SUMO-1 both *in vitro* and *in vivo*, resulting in dynamic regulation of the promoter-binding activity of TFIID [32]. The endoribonuclease Dicer, which cleaves the stem-loop structure from pre-miRNAs, allowing them to dissociate into their mature, 20–22-nucleotide single-stranded form, has also been shown to be SUMOylated [33]. Moreover, as shown in Table 1, a number of ribosomal proteins and heat shock proteins (HSPs) have been identified as SUMOylation substrates. It is worth mentioning that several ribosomal proteins and HSPs isolated from human sperm have also been found to be SUMOylated [34]. SUMOylation of the human ribosomal protein S3 (rpS3) increases its stability [35]. In addition, HSP27 has been reported to be associated with the SUMOylation system [36,37,38,39].

In this study, recombinant baculovirus of *B. mori* nucleopolyhedrovirus (BmNPV) was used to express eGFP-sumo plus BmUbc9. *Baculoviridae* is a family of enveloped, double-stranded DNA (81.7–178.7 kb) viruses that infect invertebrates, particularly insects of the order *Lepidoptera*. Numerous reports have confirmed that the viral infection is closely associated with SUMOylation [40,41]. It is known that some viral proteins such as the chicken embryo lethal orphan (CELO) adenovirus Gam1 protein [42,43], the human papillomavirus (HPV) E6 protein [44,45], and the Epstein-Barr virus (EBV) latent membrane protein 1 (LMP1) [46] can target sumoylation enzymes, causing broad effects on host SUMO substrates. In addition, some viral proteins such as the human cytomegalovirus (HCMV) IE1 protein [47] and the HPV E1 protein [48] can exploit the host sumoylation mechanism because they need to be SUMO-modified in order to exert their functions. Some other viral proteins such as human herpes virus-6 (HHV6) IE1, Epstein-Barr virus (EBV) Zta, EBV Rta, human immunodeficiency virus (HIV) P6 Gag, and the N protein of severe acute respiratory syndrome coronavirus (SARS-CoV) have all been shown to take advantage of the host sumoylation system [5,49,50]. In addition, some viruses encode sumoylation mimicking enzymes, such as the Kaposi’s sarcoma-associated herpes virus (KSHV) K-bZIP protein and the adenovirus early region 1B 55-kDa protein (E1B-55K) protein, both of which possess SUMO ligase activities [51,52]. During our investigations, several proteins from BmNPV were also identified (Table S2). It has been reported that infection of Spodoptera frugiperda (Sf9) cells by *Autographa californica* multiple nuclear polyhedrosis virus (AcMNPV) leads to a decrease in the amount of free SUMO, coupled with an increase in the amount of SUMO-conjugated products [53]. Further studies will focus on the additional roles that sumoylation may be playing during baculovirus replication.

To identify the isolated SUMOylation substrates conclusively, the sequence of each protein was analyzed using the GPS-SUMO program with the threshold MEDIUM [54,55]. Of the 203 proteins identified with one or more peptides, ~74% contained at least one SUMOylation site, and ~13% contained at least one SUMO-interaction Motifs (SIM) (Table S3). An analysis of all the identified sumoylation sites showed that approximately 41% (400 out of 983 sites) of them do not conform to the ΨKxE motif. In this regard, the current understanding of sumoylation recognition is still inadequate [44]. Thus, it is possible that, although no canonical SUMOylation sites and SIMs have been identified in the remaining 13% proteins, their presence cannot be conclusively nullified.

## 3. Experimental Section

### 3.1. Plasmid Construction and Cell Transfection

The *Bmsumo* gene was amplified with the forward primer BmsumoF (5'-GTCGACgctgatgaaaagaaggga-3') and the reverse primer BmsumoR (5'-CTGCAGttactcctccggtctgctg-3'). The *BmUbc9* gene was amplified with primers UBC9FA (5'-ctcgagATGTCAGGGATAGCAAGT-3') and UBC9RA (5'-GCATGCGATATTTACTCAGCAGCA-3'). Subsequently, the *Bmsumo* and *BmUbc9* fragments were cloned into a modified pFastBac Dual vector (Invitrogen, Carlsbad, CA, USA), which contains the eGFP coding region under the polyhedrin promoter, and were then transferred into a wild type bacmid DNA by homologous recombination to construct the recombinant baculovirus bacmid eGFP-Bmsumo + BmUbc9*.* After white-blue plaque selection, the positive colonies were selected and analyzed by PCR with M13 universal primers. The empty vector was used as a negative control.

The recombinant bacmid was transfected into BmN cells for amplification. The third-generation virus (MOI = 10 pfu/cell) was further used to infect BmN cells for subsequent protein expression.

### 3.2. Protein Extraction and Immunoprecipitation

For immunoprecipitation experiments, 2 × 10^8^ cells were infected with bacmid eGFP-Bmsumo + BmUbc9 or with the “control” bacmid. At 72 h after infection, the cells were collected and washed with ice-cold PBS (pH 7.4). GFP immunoprecipitations were performed using μMACS GFP Isolation Kit (Miltenyi Biotec, Bergisch Gladbach, Germany), according to the manufacturer’s instructions. In brief, cells were lysed with 1 mL ice-cold lysis buffer (150 mM NaCl, 1% Triton X-100, 50 mM Tris–HCl (pH 8.0), 10 mM *N*-Ethylmaleimide (NEM) (Sigma-Aldrich, Shanghai, China), and 1× protease inhibitor cocktail (Roche, Shanghai, China)) for 1 h on ice. After centrifugation for 10 min at 10,000× g, the cell supernatants were collected and incubated with 50 μL anti-GFP MicroBeads for 30 min on ice, with gentle shaking. The incubated mixtures were then applied onto the column and washed with 10× 200 μL washing buffer 1 (150 mM NaCl, 1% NP-40, 0.5% sodium deoxycholate, 0.1% SDS, 50 mM Tris–HCl (pH 8.0), 10 mM NEM, and 1× protease inhibitor cocktail). After rinsing the column with 200 μL washing buffer 2 (20 mM Tris–HCl, pH 7.5), 20 μL of preheated (95 °C) elution buffer (50 mM Tris–HCl (pH 6.8), 50 mM DTT, 1% SDS, 1 mM EDTA, 0.005% bromophenol blue, and 10% glycerol) was loaded onto the column and incubated for 5 min at room temperature. Another 50 μL of preheated (95 °C) elution buffer was applied onto the column, and the eluates were collected for SDS-PAGE and western analysis.

### 3.3. Western Blotting

Aliquots of each input lysate and both the eGFP-Bmsumo + BmUbc9 and control preparations were subjected to SDS-PAGE. After electrophoresis, the protein samples were transferred onto a PVDF membrane (Immobilon-P, Millipore, Merck KGaA, Darmstadt, Germany) in cold Towbin buffer (0.025 M Tris, 0.19 M Glycine, and 20% methanol) by using the Trans-Blot Cell apparatus (Bio-Rad, Shanghai, China). After blocking with 5% skimmed milk in PBS-T (1× PBS and 0.1% Tween-20), the membrane was washed three times with PBS-T for 5 min and was incubated with either the eGFP-epitope (Beyotime, Beijing, China) antibody or in PBS-T with 5% skimmed milk at 37 °C for 1 h. Subsequently, either anti-rabbit or anti-goat HRP-conjugated secondary antibodies (Pierce, Rockford, IL, USA) were utilized to detect the reactive band(s). The results were visualized with the ECL detection system (Amersham Biosciences, Piscataway, NJ, USA).

### 3.4. Antibody Preparation and Confocal Laser-Scanning Microscopy

The *BmSUMO* fragment was gel purified and subcloned into the expression vector pET28a with a 6× His tag at its *N*-terminus. The fused protein was expressed in *E. coli* BL21 by inducing with 1 mM Isopropyl β-d-Thiogalactoside (IPTG) at 30 °C. The polyclonal antibody was raised using standard procedures. The purified SUMO protein was mixed with complete Freund’s adjuvant and injected into New Zealand White rabbits, followed by two injections in incomplete Freund’s adjuvant.

The BmN cells were washed three times with 1× PBS and fixed in methanol:acetone (1:1) on ice for 15 min; this was followed by three washes with 1× PBS. The cells were then incubated with BmSUMO polyclonal antibody for 1 h at room temperature. After washing, the cells were incubated with Protein-G fused with enhanced green fluorescent protein (eGFP) and stained with the nucleus (DNA)-specific stain DAPI (Sigma, Shanghai, China) for 1 h. The cells were directly observed using a Leica TCS SP5 confocal laser-scanning microscope (Leica, Shanghai, China). The control was prepared as described above, except that the antibody used was pre-immune serum.

### 3.5. LC–ESI-MS/MS Analysis

After adjusting the pH to 8.5 with 1 M ammonium bicarbonate, the total protein (100 μg) extracted from each sample was chemically reduced for 1 h at 60 °C by adding dithiothreitol (DTT) at a final concentration of 10 mM and was carboxyamidomethylated in 55 mM iodoacetamide for 45 min at room temperature in the dark. Then, Trypsin Gold (Promega, Madison, WI, USA) was added to a final substrate/enzyme ratio of 30:1 (*w*/*w*). Trypsin digestion was carried out at 37 °C for 16 h. After digestion, the peptide mixture was acidified by 10 μL of formic acid for MS analysis. Each peptide sample was desalted using a Strata X column (Phenomenex, Guangzhou, China), vacuum-dried, and then resuspended in 200 μL of buffer A (2% acetonitrile and 0.1% formic acid). After centrifugation at 20,000× *g* for 10 min, the supernatant was recovered to obtain a peptide solution with a final concentration of ~0.5 μg/μL. In total, 10 μL of the supernatant was loaded onto a 2-cm C18 trap column in a LC-20AD nanoHPLC (Shimadzu, Kyoto, Japan) by using the autosampler. Then, the peptides were eluted onto an in-house packed 10-cm analytical C18 column (inner diameter, 75 μm). The samples were loaded at 8 μL/min for 4 min. Then, a gradient of 2% to 35% buffer B (98% CAN and 0.1% FA) was run for 44 min at 300 nL/min, followed by a 2-min linear gradient to 80% buffer B, and maintenance at 80% buffer B for 4 min, following which it was finally adjusted to 5% buffer B in 1 min. The peptides were subjected to nanoelectrospray ionization followed by tandem mass spectrometry (MS/MS) in a QEXACTIVE (ThermoFisher Scientific, San Jose, CA, USA) coupled online to the HPLC. Peptides were detected in the Orbitrap at a resolution of 70,000. The peptides were selected for MS/MS using high-energy collision dissociation (HCD) operating mode with a normalized collision energy setting of 27.0; ion fragments were detected in the Orbitrap at a resolution of 17,500. A data-dependent procedure that alternated between one MS scan followed by 15 MS/MS scans was applied for the 15 most abundant precursor ions above a threshold ion count of 20,000 in the MS survey scan with a dynamic exclusion duration of 15 s. The electrospray voltage applied was 1.6 kV. Automatic gain control (AGC) was used to optimize the spectra generated by the Orbitrap. The AGC target for full MS was 3 × 10^6^ and 1 × 10^5^ for MS2. For MS scans, the *m*/*z* scan range was 350 to 2000 Da. For MS2 scans, the *m*/*z* scan range was 100–1800.

### 3.6. Confirmation of SUMOylated Proteins in S2 Cells

Flag-tagged SUMO was amplified by primers DrBmSUMO F (5'-cgGAATTCatggattacaaggatgagacgataaggctgatgaaaagaaggga-3') and DrBmSUMO R (5'-ccCTCGAGttacactagggacactcct-3'). The BGIBMGA001549 was amplified by primers BGIBMGA001549F (5'-cgGAATTCatgcagatctttgtgaaaactcta-3') and BGIBMGA001549R (5'-ccCTCGAGcccattcctcctcgtaga-3'). BGIBMGA000511 was amplified by primers BGIBMGA000511F (5'-cgGAATTCatgtttaagggcctagtagta-3') and BGIBMGA000511R (5'-ccCTCGAGaggctgcatcctgagg-3'). BGIBMGA004023 was amplified by primers BGIBMGA004023F (5'-cgGAATTCatgaaagacttagttgacgta-3') and BGIBMGA004023R (5'-ccCTCGAGcaaaggttccggttcacga-3'). BGIBMGA006751 was amplified by primers BGIBMGA006751F (5'-cgGAATTCatggcagatgatatgcccaca-3') and BGIBMGA006751R (5'-ccCTCGAGtaagtcttcatcttcctca-3'). All PCR fragments were inserted into pMT/V5-His B vector (Invitrogen, Carlsbad, CA, USA). GFP-His construction was set as a control.

*Drosophila* S2 cells were grown in Schneider’s insect medium (Sigma-Aldrich, Shanghai, China) supplemented with 10% FBS. 1 × 10^7^ of S2 cells were transfected with 1 µg of each SUMO and SUMOyalation candidate constructions. Twenty-four hours after transfection, 0.5 mM CuSO_4_ was added to the medium to induce protein expression. Forty-eight hours after induction, cells were collected and washed with PBS. Then washed cell were lysed in IP buffer (25 mM Tris–HCl pH 7.4, 150 mM NaCl, 1 mM EDTA, 1% NP-40 and 5% glycerol) supplemented with 10 mM *N*-ethylmaleimide and 1× protease inhibitor cocktail on ice for 1 h. After 12,000 rpm/10 min centrifugation, supernatants were collected and incubated with Anti-FLAG M2 Magnetic Beads (Sigma-Aldrich, Shanghai, China) on ice for 3 h. The beads were finally collected and washed three times with immunoprecipitation buffer. Proteins binding to the beads were eluted by adding 20 µL of 1× electrophoresis sample buffer and analyzed by Western-blotting with Anti-His antibody (Sigma-Aldrich, Shanghai, China). The whole cell lysates were used as input samples.

### 3.7. Data Analysis

Raw data files acquired from the Orbitrap were converted into MGF files using Proteome Discoverer 1.2 (PD 1.2, Thermo, Pittsburgh, PA, USA), and the MGF files were searched. Protein identification was performed using the Mascot search engine (Matrix Science, London, UK; version 2.3.02) against a database containing 21,893 sequences. For protein identification, a mass tolerance of 20 ppm was permitted for intact peptide masses and that of 0.6 Da for fragmented ions, with allowance for one missed cleavage in the tryptic digests. Gln->pyro-Glu (*N*-term Q), Oxidation (M), and Deamidated (NQ) were the potential variable modifications, and Carbamidomethyl (C) was the fixed modification. The charged states of the peptides were set to +2 and +3 specifically, and an automatic decoy database search was performed in Mascot by choosing the decoy checkbox in which a random sequence of database is generated and tested for raw spectra as well as the real database. To reduce the probability of false peptide identification, only peptides with significant scores (≥20) at the 99% confidence interval generated by a Mascot probability analysis greater than “identity” were counted as identified. Every confirmatory protein identification involved at least one unique peptide. Functional annotations of the proteins were conducted using Blast2GO program against the non-redundant protein database (NR; NCBI). The KEGG database [56,57] and the COG database [58,59] were used to classify and group these identified proteins.

## 4. Conclusions

Sumoylation represents a vital PTM that pervades numerous aspects of cell biology, including protein targeting, transcriptional regulation, signal transduction, and cell division. To provide further insight into this complex process, a proteomics approach was undertaken to identify the targets of SUMOylation in the model lepidopteran *B. mori*. A total of 68 candidate SUMOylated proteins were identified from the *B. mori* proteome, of which 59 proteins were functionally categorized to GO terms. KEGG pathways analysis showed that 46 proteins were involved in 76 pathways that mainly play a role in metabolism, spliceosome and ribosome functions, and in RNA transport. In total, 74% of the identified proteins were predicted to have at least one SUMOylation site. These data shed light on the crucial process of protein sumoylation in *B. mori*.

## Supplementary Materials

Supplementary tables can be found at http://www.mdpi.com/1422-0067/15/12/22011/s1.
